# Modulation of Wnt signaling is essential for the differentiation of ciliated epithelial cells in human airways

**DOI:** 10.1002/1873-3468.12851

**Published:** 2017-10-10

**Authors:** Andreas Schmid, Juliette Sailland, Lisa Novak, Nathalie Baumlin, Nevis Fregien, Matthias Salathe

**Affiliations:** ^1^ Division of Pulmonary, Allergy, Critical Care and Sleep Medicine University of Miami School of Medicine FL USA; ^2^ Department of Cell Biology University of Miami School of Medicine FL USA

**Keywords:** airway epithelium, ciliated cells, Dickkopf, differentiation, Wnt signaling

## Abstract

Wnt signaling is essential for the differentiation of airway epithelial cells during development. Here, we examined the role of Wnt signaling during redifferentiation of ciliated airway epithelial cells *in vitro* at the air liquid interface as a model of airway epithelial repair. Phases of proliferation and differentiation were defined. Markers of squamous metaplasia and epithelial ciliation were followed while enhancing β‐catenin signaling by blocking glycogen synthase kinase 3β with SB216763 and shRNA as well as inhibiting canonical WNT signaling with apical application of Dickkopf 1 (Dkk1). Our findings indicate that enhanced β‐catenin signaling decreases the number of ciliated cells and causes squamous changes in the epithelium, whereas treatment with DDk1 leads to an increased number of ciliated cells.

## Abbreviations

**ALI**, air liquid interface

**BrdU**, 5‐bromo‐2′‐deoxyuridine

**Dkk1**, Dickkopf 1

**FOXJ1**, forkhead box 1

**Fz, frizzled**

**GSK3β**, glycogen synthase kinase 3β

**KD**, knock down

**LRP5/6**, low‐density lipoprotein receptor‐related proteins 5 or 6

**MCC**, mucociliary clearance

**NHBE**, normal human bronchial epithelium

**PCP**, planar cell polarity

**PFA**, paraformaldehyde

**TCF**, T cell factor

Similar to skin, the airway epithelial surface is directly exposed to the environment. The airway surface can be injured by inhalation of dust, cigarette smoke, and infectious agents. Chronic exposure to irritants causes squamous metaplasia, goblet and basal cell hyperplasia, and atrophy of the epithelium 1, 2. However, the airway epithelium has the ability to repair damage by proliferation and differentiation of epithelial progenitor cells 3, 4.

Mucociliary clearance (MCC) is a key mechanism for protecting the airways from inhaled irritants 5. MCC is a two‐component system that requires mucus to trap inhaled substances and beating cilia to propel the mucus out of the airways. The pseudostratified airway epithelium contains all cell types needed for effective MCC. Repopulation of the epithelium with ciliated cells is a critical part of airway epithelial repair. Multiciliated cells are covered apically with cilia. The ciliary length, beat frequency, and directionality along the tissue axis are strictly regulated. Motile airway cilia are oriented in a common direction by planar cell polarity (PCP) signaling. PCP is determined by a cell–cell communication via PCP complexes and polarizes all cells with respect to the proximal‐distal tissue axis to establish molecular asymmetry by core proteins that segregate distal (Frizzled, Dishevelled, Diego, and Flamingo) and proximal (Van Gogh Like and Prickle) 6, 7.

The genesis of cilia is a complex process. It involves apical organization of an actin network 8 and basal body multiplication. Basal bodies dock to the apical actin web 9. This is followed by building cilia from the base of the basal bodies. FOXJ1 is necessary for ciliogenesis 10, 11 and orchestrating ciliary differentiation together with regulatory factor X transcription factors 12. However, the fate determination of progenitor cells to become ciliated cells occurs prior to FOXJ1 expression and is an important step in the repopulation of ciliated airway epithelial cells 4. The transcription factor MYB has recently been described as one of the key factors for multiciliary cell fate determination. Myb acts upstream of foxj1 in mice and is essential for centriole amplification during differentiation of multiciliated airway cells 13, 14. Multicilin (Mcidas) forms a complex with E2f4 or E2f5 and Dp1, which activates gene expression that is required for basal body assembly during multiciliated cell differentiation 15 and acts upstream of Myb 14. Interestingly, MYB expression is increased in airway epithelia of patients with chronic airway disease 13.

Wnt signaling is important for embryonic development 16, 17 and regulation of cell proliferation and differentiation 18. However, it affects many disease processes 19, 20, 21 and plays an important role in wound healing 22, 23 and airway epithelial repair 24. Wnt also regulates foxj1 expression in Zebrafish 25 and *Xenopus* 26. Furthermore, McCauley *et al*. 27 recently demonstrated a major role of Wnt signaling in airway cell differentiation: cyclical modulation of canonical Wnt signaling enables rapid directed differentiation of human induced pluripotent stem cells (iPSCs) via NKX2‐1+ into functional proximal airway organoids. In humans, nineteen Wnt proteins and ten Frizzled (Fz) membrane receptors initiate Wnt signaling. Canonical and noncanonical pathways have been described. The canonical pathway is defined by secreted Wnt peptides that bind to membrane bound Fz receptors, which are associated with their coreceptors, Low‐density lipoprotein Receptor‐related Proteins 5 or 6 (LRP5/6), disheveled and Axin 19, 28. Canonical signaling increases cytoplasmic β‐catenin by inactivating glycogen synthase kinase 3β (GSK3β) as part of the β‐catenin degrading complex. β‐catenin enters the nucleus and heterodimerizes with a transcription factor T cell factor (TCF) to induce transcription of canonical target genes that generally promote proliferation 19. The noncanonical pathway is defined by Wnt peptides binding to Fz receptors not bound to LRP 5/6, resulting in signaling via calcium, small GTPases, and JNK pathways 29, 30, 31.

Dickkopf‐1 (Dkk1) was identified as an inhibitor of canonical Wnt signaling 32. Dkk1 is a paracrine Wnt inhibitor that binds to LRP5/6 and blocks hetero‐dimerization with Fz receptors 33. Reduction of Dkk1 expression by siRNA upregulated the expression of β‐catenin, c‐MYC, and cyclin D1 in H7402 cells 34.

Here, we examined the effect of Wnt signal modulation by Dkk1 and inhibition of GSK3β on the differentiation of ciliated airway epithelial cells in human air liquid interface (ALI) cultures.

## Material and methods

### Chemicals

Human recombinant Dkk1 protein was purchased from R&D Systems Inc. (Minneapolis, MN, USA). All other chemicals were acquired from Sigma‐Aldrich (St. Louis, MO, USA), if not specified otherwise.

### Cell cultures

Human airways were obtained from organ donors whose lungs were rejected for transplant. Consent for research was obtained by the Life Alliance Organ Recovery Agency of the University of Miami with local IRB‐approved written consents conforming to the standards set by the Declaration of Helsinki. From these lungs, airway epithelial cells were isolated, expanded on collagen I‐coated dishes and differentiated at an ALI on collagen IV‐coated T‐clear filters with 0.4 μm pores (Costar Corning, Corning, NY, USA) as previously described 35, 36, 37, 38, 39. In all experiments, we defined day 1 as the day when cells were placed on T‐clear filters. Cells were grown until confluence (4 or 5 days) with media in both upper and lower chambers and then switched to an ALI by removing the media from the upper chamber. For all experiments, cells from at least two different lungs were used with 2 or 3 cultures per lung.

### Immunohistochemistry

Normal human airway epithelial cells grown on Transwell filters at the ALI were fixed with 4% paraformaldehyde (PFA) 40 and permeabilized with 0.1% Triton X‐100 in PBS followed by blocking with 10% nonfat dry milk in PBS. Primary antibodies against acetylated α‐tubulin, Dkk1 (Origene, Rockville, MD, USA), involucrin, and e‐cadherin (Life Technologies, Grand Island, NY, USA) were used. Secondary antibodies were fluorescently labeled with Alexa Fluor (Molecular Probes, Grand Island, NY, USA). Nuclei were stained with DAPI (KPL Inc., Gaithersburg, MD, USA).

Tracheal rings from three human lungs from nonsmokers were fixed in 4% PFA, embedded in paraffin and sectioned. Sections were processed as previously described 41. Briefly, sections were deparaffinized with xylene, rehydrated with denatured ethanol and subjected to heat‐induced antigen retrieval with 10 mm sodium citrate buffer (pH 6.0). After blocking for 1 h with gelatin 1% (wt/vol) in PBS, sections were incubated overnight at 4 °C with rabbit anti‐Dkk1 antibody or nonimmune rabbit IgG as a control (Santa Cruz Biotechnology Inc., Santa Cruz, CA, USA) before staining with Alexa 555 anti‐rabbit IgG (1 mg·mL^−1^). Cilia were labeled with mouse antiacetylated α‐tubulin antibodies (1 : 1000) and stained with Alexa 647 anti‐mouse IgG (1 mg·mL^−1^). Nuclei were visualized with DAPI and slides were mounted with Gel/Mount (Biomeda, Foster City, CA, USA).

### 5‐bromo‐2′‐deoxyuridine (BrdU) labeling

To quantify proliferation, cells were labeled with 100 μm BrdU in DPBS for 12 h before fixation with 4% PFA, treatment with 1M and 2M HCl for 10 min each at 37 °C and staining with anti‐BrdU antibodies (Santa Cruz, Biotechnology Inc) using a fluorescent secondary Alexa antibody (Molecular Probes). Proliferation index was determined as the ratio of nuclei stained with anti‐BrdU and total nuclei stained with DAPI.

### Western blotting

Total cell protein was prepared from ALI cultured cells by lysis with 1% SDS in 10 mm Tris, pH 8.5, and 0.1 mm EDTA in the presence of protease inhibitors and cleared from debris by centrifugation. Ten microgram of protein was separated using SDS/PAGE gels (Bio‐Rad, Hercules, CA, USA) and electro‐blotted onto Immobilon P membranes (Millipore, Billerica, MA, USA). The membranes were blocked with 5% nonfat dry milk in PBS and incubated with the primary antibody for 2 h at room temperature, washed with PBS and 0.05% Tween and incubated with a secondary horseradish peroxidase‐labeled antibody (KPL Inc., Gaithersburg, MD, USA). Signal was detected and quantified by chemiluminescence (Pierce, Rockfort, IL, USA) on a ChemiDoc XRS system (Bio‐Rad). Mouse anti‐β‐actin (Abcam, Cambridge, MA) was used for loading control. The signals were quantified using image lab software (Bio‐Rad).

### Quantitative RT‐PCR

Total RNA was isolated from ALI cultures using RNeasy mini kit (Qiagen, Valencia, CA, USA). RNA was quantified utilizing a NanoDrop 1000 (Thermo Scientific, Rockford, IL, USA). cDNA was made from total RNA using the iScript Kit (Bio‐Rad). mRNAs of interest were quantified by real‐time quantitative RT‐PCR using a Bio‐Rad iCycler and TaqMan gene expression assays (Life Technologies). The following assays were used: GAPDH (4352934E), Cyclin D1 (Hs00765553_m1), β‐catenin (Hs00355049_m1), FOXJ1 (Hs00230964_m1), Wnt4 (Hs00229142_m1), Wnt7a (Hs01114990_m1), GSK3β (Hs01047719_m1), involucrin (Hs00846307_m1), and E‐cadherin (Hs01023894_m1). Changes in expression were calculated using the ΔΔ*C*_t_ method or as a fraction of GAPDH expression as $2^{-\Delta C_{t}}$ × 1000.

### shRNA virus production

Lentiviral shRNA targeting GSK3β (RHS4531) and a nontargeting control vector (RHS4349) were bought from Open Biosystems (Lafayette, CO, USA). Virus was used as described 42.

### Statistics

Results were compared by one‐way ANOVA and, if a significant difference was found, a parametric or nonparametric analysis for comparison of individual groups was done, for which significance was reported in the figure legends. For some of the measures, t‐tests were used to compare groups as indicated in the figure legends. prism 5 (GraphPad, La Jolla, CA, USA) was used for statistical calculations. *P* < 0.05 was accepted as significant.

## Results

### Time course of proliferation and differentiation of NHBE cells at the ALI

**Figure 1:**
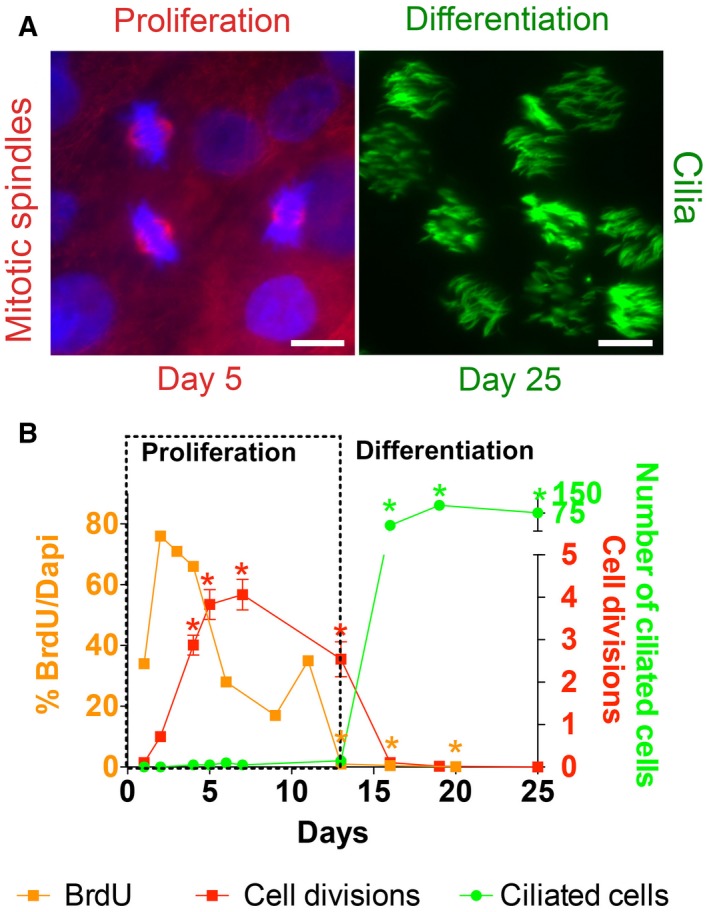
Cell proliferation precedes ciliated cell differentiation. During differentiation of airway epithelial cells *in vitro*, a phase of cell proliferation precedes a phase of differentiation. (A) Left panel: Representative picture of mitotic spindles used for quantification. Immunofluorescent staining of mitotic spindles (acetylated α‐tubulin, red) indicating proliferating cells on day 5. Nuclei are labeled with DAPI (blue). Right panel: immunofluorescent staining of motile cilia (acetylated α‐tubulin, green) in differentiated cells on day 25. Bars 5 μm. (B) Quantification of BrdU positive nuclei as a percentage of nuclei stained with DAPI (orange squares, left *y*‐axis), mitotic spindles per visual field (red squares, lower right *y*‐axis), and ciliated cells per visual field (green dots, upper right *y*‐axis) during differentiation. These data demonstrate an early phase of proliferation (outlined by dotted lines), followed by a phase of differentiation to ciliated cells. Comparisons of each time course were made using one‐way ANOVA and Tukey's multiple comparison test with accepted significance level (*P < 0.05).

To study the differentiation of human airway epithelial cells, undifferentiated normal human bronchial epithelium (NHBE) cells were seeded onto Transwell filters (defined as day 0) and proliferated for 4–5 days submerged before media were removed from the upper chamber to initiate the ALI. We evaluated proliferation using two methods. First, mitotic activity was assessed by counting nuclei with mitotic spindles, visualized by immunofluorescent staining for acetylated α‐tubulin. Counting the number of mitotic spindles (*n* = 50 visual fields 40× for each time point from two different lungs), averages of 2.9 ± 0.2, 3.8 ± 0.4, 4.1 ± 0.4, and 2.5 ± 0.4 mitoses per field were found on days 4, 5, 7, and 13, respectively, but none on days 16, 19, or 25 (Fig. 1, *P* < 0.05). Proliferation was also characterized by BrdU incorporation (Fig. 1). Staining for BrdU (*n* = 50 visual fields 40× from 2 different lungs) peaked with 66–76% BrdU positive nuclei compared to DAPI stained per visual field on days 2, 3, and 4. Days 13, 16, and 20 had a significantly lower BrdU staining with 1%, 0.5%, and 0.1%, suggesting there is little cell proliferation at this time. To gauge the time course of differentiation, the ciliated cell population was examined by staining for cilia‐enriched acetylated α‐tubulin at the apical surface and counting ciliated cells per visual field. Ciliated cells were detected on day 16 and remained present until day 25 with 27.9 ± 3 vs 105.4 ± 11.5 vs 76.5 ± 9.4 for days 16, 20, and 25, significantly increased compared to the previous days (Fig. 1; *n* = 20 from two different lungs, *P* < 0.05).

These data suggest that NHBE cells go through two different phases during differentiation: a proliferative first phase with an increase in cell number during the first 2 weeks of culture and a second phase with ciliated cell differentiation with little cell proliferation after 16 days in culture.

### Wnt expression and signaling during proliferation and differentiation of NHBE cells

**Figure 2:**
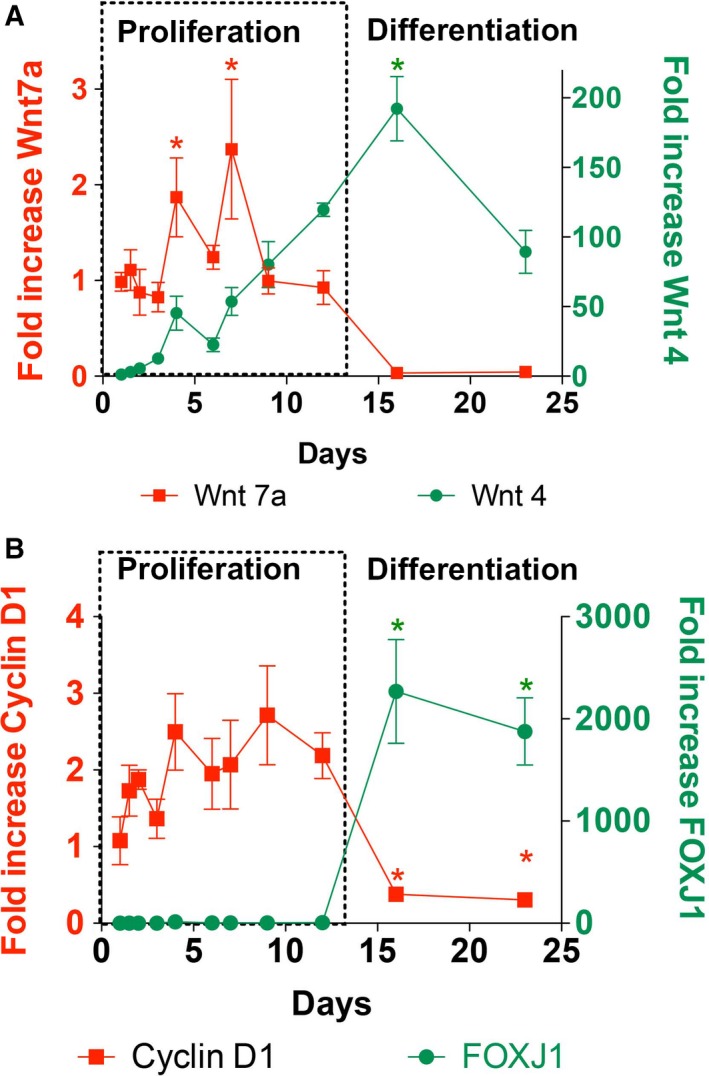
Wnt signaling during proliferation and differentiation. (A) Quantitative RT‐PCR of Wnt7a (red squares, left *y*‐axis) and Wnt4 (green dots, right *y*‐axis) mRNAs during NHBE cell differentiation shows higher Wnt7a expression during proliferation and lower expression during differentiation. One‐way ANOVA and Tukey's multiple comparison test showed significantly increased levels of Wnt7a on day 4 and 7 compared to levels on days 2, 3, 16, and 23 (*P < 0.05). Wnt4 expression was lower during proliferation and increased during differentiation. Levels on day 16 were significantly higher than all levels from day 1 to 7 using one‐way ANOVA and Tukey's multiple comparison test (*P < 0.05). (B) mRNA expression of Cyclin D1, a canonical Wnt target gene (red squares, left *y*‐axis), and FOXJ1, a marker of ciliated cell differentiation (green circles, right *y*‐axis), during NHBE cell differentiation. FOXJ1 expression was significantly increased on days 16 and 23 compared to all previous time points using one‐way ANOVA and Tukey's multiple comparison test (*P < 0.05). Cyclin D1 levels on days 16 and 23 were significantly decreased compared to days 4 and 9, again using one‐way ANOVA and Tukey's multiple comparison test (*P < 0.05). The collection on day 1 (24 h after placing on filters) was set as 1 for fold increased calculations.

As there are 19 different Wnt genes, microarray gene expression data during mucociliary differentiation were examined (Geo Dataset 2615; http://www.ncbi.nlm.nih.gov/gds/?term=GDS2615) 43 to determine the Wnt genes expressed in airway epithelial cells and their patterns of expression. These data showed that Wnt7a mRNA expression decreased and Wnt4 increased as cultures differentiated. We confirmed these findings in our cells by qRT‐PCR. Wnt7a expression was higher during the early, proliferative phase and decreased by day 16 when ciliated cells appeared. It remained low during the remainder of the differentiation phase (*n* = 3 cell cultures from three different lungs; Fig. 2 red squares). In contrast, Wnt4 mRNA expression was lower during the proliferative phase and increased about 100–200 times during the differentiation phase (*n* = 3 from three different lungs, Fig. 2).

β‐Catenin is a key downstream effector of the Wnt pathway. Stabilization of β‐catenin results in increased expression of target genes that drive cell proliferation such as Cyclin D1. The expression of Cyclin D1 mRNAs was measured by qRT‐PCR and found to be higher during the proliferative culture phase of NHBE cells but significantly decreased on day 15 and 23 (*n* = 3 from three different lungs, Fig. 2B). Conversely, FOXJ1 mRNA, a marker gene of ciliogenesis 11, significantly increased on day 16 and 23 in culture (*n* = 3 from three different lungs; Fig. 2).

These data suggest that Wnt7a/β‐catenin signaling may be involved in the early proliferation phase while Wnt4 signaling may be important for differentiation to the ciliated phenotype.

### Role of β‐catenin during differentiation

**Figure 3:**
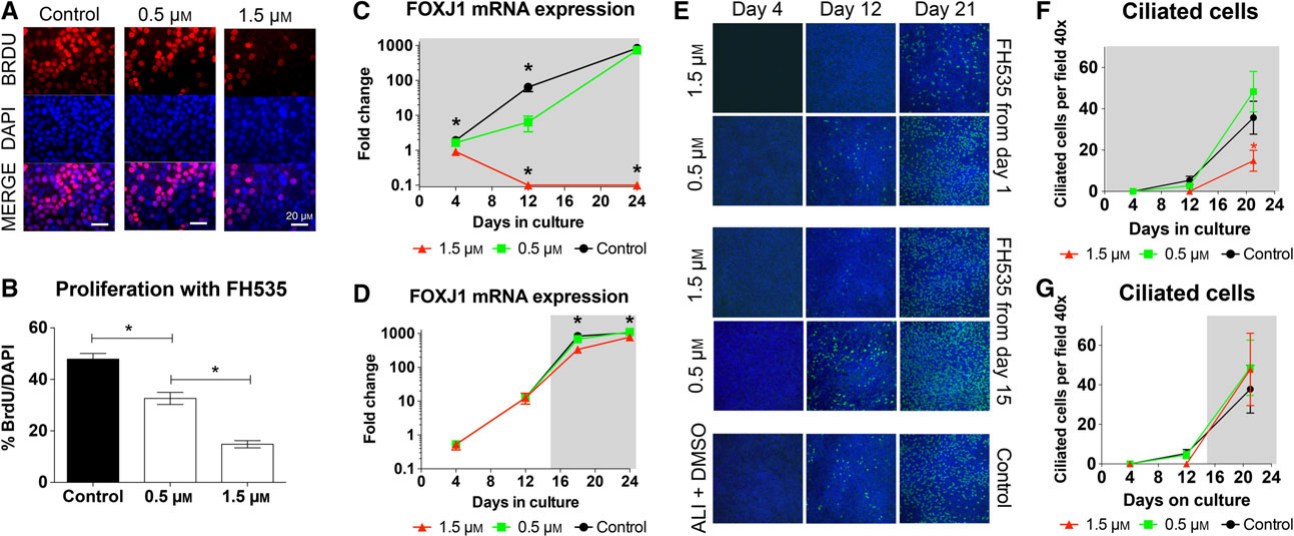
β‐catenin signaling is required for proliferation, but not for differentiation. (A) Immunofluorescence of NHBE cells labeled with BrdU (red, top panels) on the fifth day of culture in the presence of the β‐catenin inhibitor FH535 at 0 μm (control), 0.5 μm or 1.5 μm. Nuclei are labeled with DAPI (blue, middle panels). (B) Quantification of BrdU incorporation shown in panel A revealed a significant, concentration‐dependent decrease of proliferation with FH535 (*n* = 45, from 3 lungs) using one‐way ANOVA and Tukey's multiple comparison test (*P < 0.05). (C) qRT‐PCR of FOXJ1 mRNA during differentiation of NHBE cells treated with 0 μm (control, black circles), 0.5 μm (green squares) or 1.5 μm (red triangles) FH535 starting on day 0 (indicated by gray background). Cultures treated with 0.5 μm FH535 lagged behind in FOXJ1 expression on day 12, but caught up with the control cultures on day 24 (*n* = 9 from three lungs) using one‐way ANOVA and Tukey's multiple comparison test (*P < 0.05). (D) qRT‐PCR of FOXJ1 mRNA in NHBE cells treated with 0 μm (control, black circles), 0.5 μm (green squares) or 1.5 μm (red triangles) FH535 during the differentiation phase only, beginning on day 15 until day 24 (indicated by gray background). Addition of FH535 after the proliferation showed decreased FOXJ1 expression on days 18 and 24 at 1.5 μm FH535 compared to the control (*n* = 9, from 3 lungs), while 0.5 μm had no effect using one‐way ANOVA and Tukey's Multiple Comparison test (*P < 0.05). (E) Demonstration of ciliated cells in cultures treated with FH353 from day 1 (upper panel) and from day 15 (middle panel) and untreated control (lower panel) at different time points (cilia green, DAPI blue). (F) Treatment with 1.5 μm FH535 decreased the ciliated cell counts at day 21 compared to treatment with 0.5 μm FH535 and DMSO control (*n* = 6, from two lungs) using one‐way ANOVA and Tukey's multiple comparison test (*P < 0.05). (G) There was no difference in the number of ciliated cells on day 12 and 21when FH535 was added on day 1 or 15 days after plating the cells compared to control.

To further investigate the role of β‐catenin signaling during NHBE proliferation and differentiation, cells were treated throughout differentiation (day 1–24) with FH535, a nuclear Wnt/β‐catenin and PPARγ signaling inhibitor 44. Proliferation was measured by BrdU incorporation on day 5 (Fig. 3A,B) and showed a significant decrease compared to DMSO control conditions (*P* < 0.05), using two different concentrations (*n* = 45 from three different lungs). Addition of 0.5 μm FH535 decreased proliferation by ~ 32% (47.8% vs 32.6% BrdU positive cells) and addition of 1.5 μm FH535 decreased it by ~ 69% (47.8% vs 14.8% BrdU positive cells). To determine the effect of FH535 on ciliated cell differentiation, the level of FOXJ1 mRNA was measured on days 4, 12, and 24. Blocking β‐catenin with 1.5 μm FH535 resulted in this experiment in cell death between day 4 and 12. Cells treated with 0.5 μm FH535 had a lower level of FOXJ1 mRNA expression on day 12 (ratio of FOXJ1/GAPDH 6.4 ± 3.02 vs 64.2 ± 16.3 fold increase in control cells; *n* = 9 from three different lungs, *P* < 0.05), but a similar level on day 24 (734.3 ± 102.9 vs 833.4 ± 108.9 fold increase in control cells, *n* = 9, *P* > 0.05; Fig. 3). Similar results were obtained when directly measuring cilia by staining for acetylated tubulin (Fig. 3F). When adding FH535 from the day the cells were plated on filters, the differences on day 12 between DMSO control vs 0.5 μm vs 1.5 μm FH535 were 3.55 ± 1.5 vs 1.8 ± 0.9 vs 0 ciliated cells per 40× visual field. However, on day 21 there were 35.7 ± 7.8 vs 32.2 ± 10.2 vs 9.9 ± 4.1 ciliated cells per 40× visual field for DMSO control vs 0.5 μm vs 1.5 μm FH535, showing a significant decrease of ciliated cells in cultures treated with 1.5 μm FH535 vs control and 0.5 μm FH535 on day 21 (*n* = 6 from two different lungs, *P* < 0.05). These results suggest that the β‐catenin pathway is linked to and modulates proliferation of NHBE cells.

To test if β‐catenin is necessary for ciliated cell differentiation, we added FH535 to the cultures only from day 15 to 25 (Fig. 3D) and measured FOXJ1 expression by qRT‐PCR and cilia presence by immunocytochemistry. In this experiment, cells treated with 0.5 μm FH535 had an undistinguishable FOXJ1 mRNA level and ciliation compared to untreated controls (fold change of FOXJ1/GAPDH of 675.5 ± 57.7 vs 834.2 ± 49 on day 18 and 1108 ± 66 vs 1050 ± 77.52 on day 24, *n* = 9, *P* > 0.05). Cells treated with 1.5 μm FH535 differentiated into ciliated cells as well, but had a decreased FOXJ1 expression on 18 days (334.7 ± 56.2 vs 836.2 ± 49, *n* = 9, *P* < 0.05) and 24 days (770 ± 42.5 vs 1050 ± 77.5, *n* = 9 from three different lungs, *P* < 0.05) compared to the control (Fig. 3D). While these changes were small, they still reached statistical significance. When ciliation was measured upon addition of FH535 on day 15, the number of ciliated cells per 40× visual field were 5.3 ± 1.9 vs 4.5 ± 1.8 vs 0 in the DMSO control vs 0.5 μm vs 1.5 μm FH535. On day 21, ciliated cells per 40× visual field were 37.8 ± 12.1 vs 45.7 ± 14 vs 47.8 ± 18.3 in control vs 0.5 μm vs 1.5 μm FH535 conditions. There was no statistically significant difference at any of the time points between the groups (Fig. 3G).

In conclusion, these results suggest that β‐catenin is required for primary human airway epithelial cells to proliferate, but not to differentiate into ciliated cells.

### Expression of Dickkopf 1 in human airways

**Figure 4:**
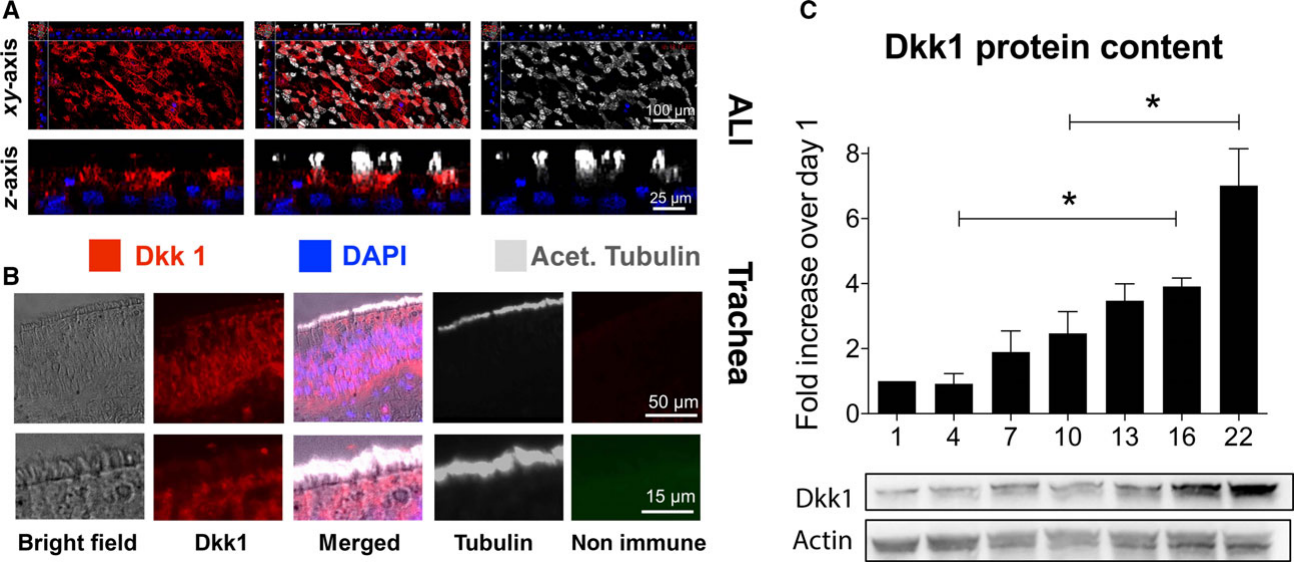
Dkk1 expression in human airway epithelial cells. (A) Confocal micrographs of differentiated ALI cultures labeled for Dkk1 (red), cilia (white) and nuclei (blue) showed Dkk1 expression. All images, but particularly z stacks, suggest localization of Dkk1 to ciliated cells. (B) In human tracheal sections from a normal individual, immunofluorescence showed the presence of Dkk1 in the airway epithelium, with expression in ciliated cells. (C) Quantification of Dkk1 protein by western blotting (normalized to actin) during differentiation of NHBE cells grown at the ALI showed a low Dkk1 expression in undifferentiated cells during the proliferative phase. A significant increase occurred during the differentiation phase using one‐way ANOVA and Tukey's multiple comparison test (*P < 0.05).

Based on the observed differential expression patterns of Wnt7a and Wnt4 during differentiation, we looked at expression of Dkk1 known to inhibit Wnt/β‐catenin signaling in the lung. Immunofluorescent staining of differentiated ALI cultures and human tracheal sections (Fig. 4) showed Dkk1 expression. During differentiation, Dkk1 protein increased significantly (Fig. 4). Compared to day 1 (set at 1), levels were not different on day 4, 7 and 10 (0.92 ± 0.31 vs 1.89 ± 0.65 vs 2.47 ± 0.67 fold of day 1) but increased significantly (*P* < 0.05) on day 16 compared to day 4 (3.91 ± 0.26 vs 0.92 ± 0.31 fold of day 1). Expression on day 22 was also significantly (*P* < 0.05) increased compared to day 10 (7.0 ± 1.14 vs 2.47 ± 0.67 fold of day 1; all *n* = 3 from three different lungs in all western blots).

These results show that Dkk1 expression is significantly increased during ciliated cell differentiation.

### Effect of Dkk1 and β‐catenin on ciliogenesis

**Figure 5:**
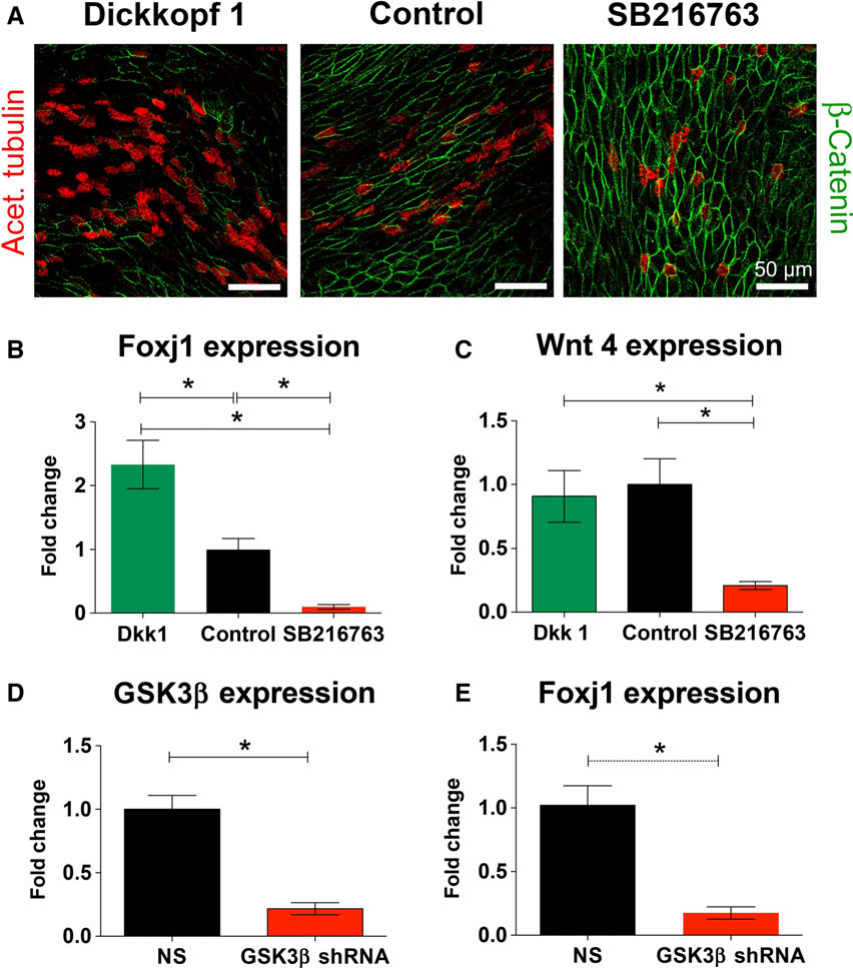
Effects of stimulating and inhibiting β‐catenin signaling on differentiation of ciliated cells. (A) Immunofluorescence showing cilia (red) and β‐catenin (green) in airway epithelial cells differentiated for 28 days in the presence of 0.1 μg·mL^−1^ Dkk1 (left), media only (center) or 10 μm of SB216763. (B, C) Quantification of FOXJ1 (B) and Wnt4 mRNA expression (C) in NHBE cells treated with Dkk1 (green bars), control (black bars) or SB216763 (red bars). These data show that inhibition of β‐catenin signaling with Dkk1 increased FOXJ1 mRNA expression with no effect on Wnt4 mRNA expression. SB216763 decreased FOXJ1 and Wnt4 mRNA expression. One‐way ANOVA and Tukey's multiple comparison test for B, C (*P < 0.05). (D) KD of GSK3β with GSK3β shRNA expressing lentiviruses (red bar) compared to a control, nontargeting construct (NS, black bar). (E) Quantification of FOXJ1 mRNA in cells transduced with GSK3β shRNA expressing lentiviruses (red bar) or a NS construct (black bar). KD of GSK3β mRNA resulted in a significant decrease in FOXJ1 expression compared to cultures infected with a NS. *T*‐test was used for D, E (*P < 0.05).

After establishing the patterns of proliferation and differentiation during airway epithelial cell differentiation at the ALI as well as associating β‐catenin signaling with the proliferative phase and Dkk1 with the differentiation phase, we asked whether a switch in Wnt signaling is necessary for ciliary differentiation. To address this question, NHBE cells were treated both apically and basolaterally for the entire 24 days of differentiation with media alone or media supplemented with SB216763 (10 μm), an inhibitor of GSK3β that stimulates β‐catenin‐associated Wnt signaling 45, or media supplemented with Dkk1 (0.1 μg·mL^−1^), a canonical Wnt signaling inhibitor 40. Only 100 μL fluid was applied on the apical side of the 1 cm^2^ Transwell filters to minimize the effect of submersion, which has been associated with impaired ciliogenesis 46, 47. After 24 days, cells were stained for acetylated tubulin to quantify ciliated cells: Dkk1 increased and SB216763 decreased the ciliated cell population compared to control cultures (Fig. 5A). This finding was paralleled by a significant increase of FOXJ1 mRNA in cultures treated with Dkk1 compared to controls (2.33 ± 0.5 fold to control) and by a decrease in SB216763‐treated cultures (0.1 ± 0.04 fold; Fig. 5B; *n* = 4 from two different lungs; *P* < 0.05). These results suggest that canonical Wnt signaling inhibits FOXJ1 expression and must be suppressed to permit ciliated cell differentiation.

Enhancing β‐catenin signaling using SB216763 also significantly decreased Wnt4 expression on day 24 (0.21 ± 0.08 fold change) compared to Dkk1‐treated cells (0.91 ± 0.2 fold change) and control cells (1 ± 0.2 fold change; *P* < 0.05; *n* = 6 from three different lungs) between SB 216763‐treated cells and other groups (Fig. 5C). While SB216763 seemed to increase the expression of Wnt7a on day 12 compared to Dkk1‐treated and control cells (1.16 ± 0.19 vs 0.89 ± 0.07 vs 1.03 ± 0.04), the changes did not reach statistical significance. There was also no difference in WNT7a expression on day 24 and in WNT4 expression on day 12.

To confirm that the results with SB216763 were related to inhibition of GSK3β and not another kinase, GSK3β knock down (KD) was performed with lentiviral‐mediated shRNA expression. qRT‐PCR of GSK3β mRNA confirmed KD in cells expressing GSK3β shRNA (0.22 ± 0.06 fold change) compared to cells expressing nontargeting shRNA (NS; 1 ± 0.13 fold change; Fig. 5D; *n* = 4 from two different lungs, *P* < 0.05). In addition, FOXJ1 mRNA expression was significantly decreased (0.18 ± 0.06 fold change) in GSK3β shRNA transduced cells vs NS cultures (Fig. 5E; *n* = 4 from two different lungs, *P* < 0.05).

In conclusion, these data suggest that increasing Dkk1 enhances differentiation of the ciliated cell population in human airway epithelial cells grown at the ALI, possibly via inhibition of the canonical Wnt7a pathway. On the other hand, stimulation of the canonical pathway via GSK3β inhibition to enhance β‐catenin signaling has the opposite effect.

### Modulation of β‐catenin signaling leads to squamous cell differentiation in the airway epithelium

**Figure 6:**
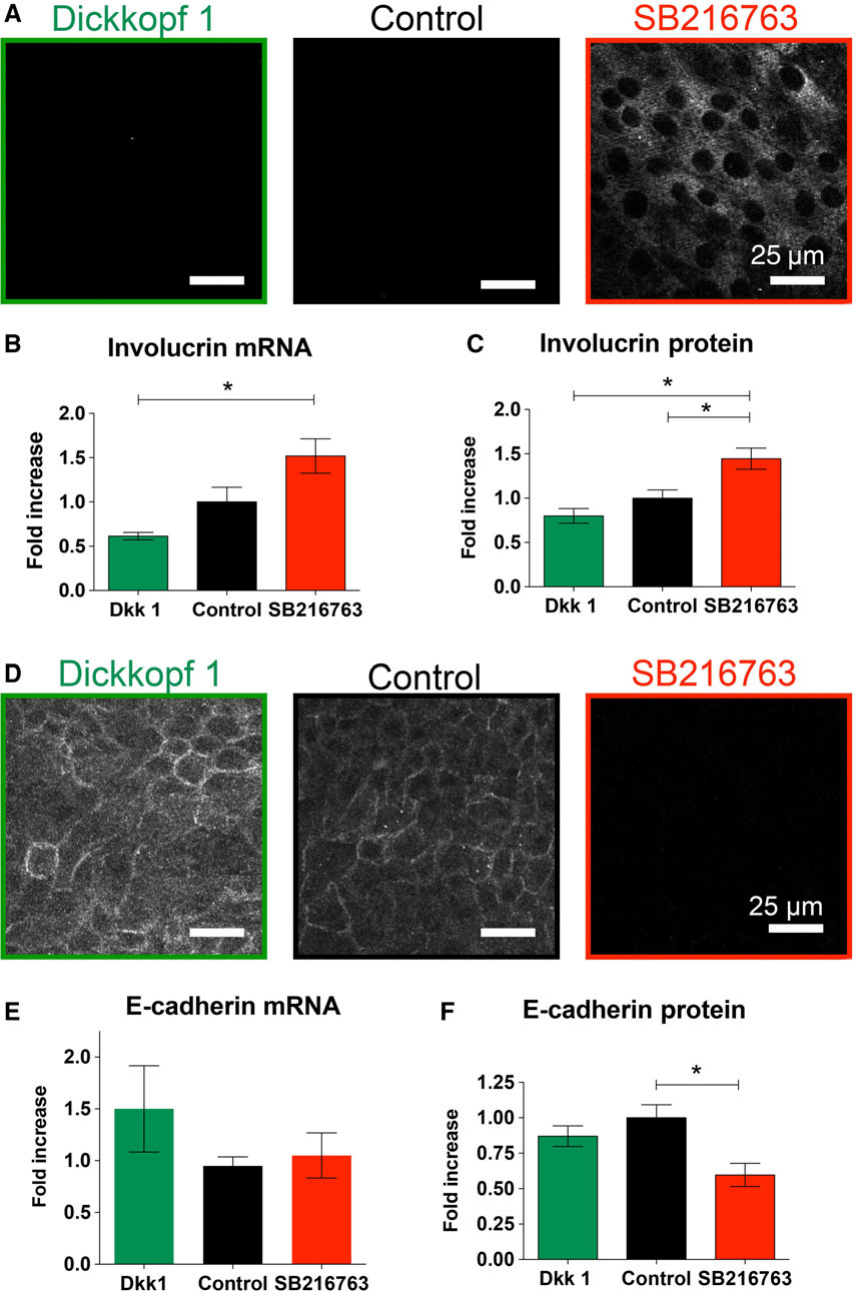
Increased β‐catenin signaling by GSK3β inhibition leads to a squamous phenotype of the airway epithelium. (A) Immunofluorescence for involucrin, a marker of squamous cells, in NHBE cells differentiated for 28 days in the presence of Dkk1 (0.1 μg·mL^−1^; left, green border), control (center, black border) or SB216763 (10 μm) (right, red border). (B, C) Quantification of involucrin mRNA by qRT‐PCR (B) and protein by western blot (C) using NHBE cells differentiated for 28 days. SB216763 treatment caused significant increases of both involucrin mRNA and protein using one‐way ANOVA and Tukey's multiple comparison test (*P < 0.05). (D) SB216763 decreased the expression of E‐cadherin as shown by immunohistochemistry. (E) No change in the mRNA expression of E‐cadherin was found in SB216763‐ and Dkk1‐treated cells, but there was decreased protein expression in SB216763‐treated cells using one‐way ANOVA and Tukey's multiple comparison test (*P < 0.05) (F).

Since manipulating canonical Wnt signaling affected ciliated cell differentiation, we examined the effect of β‐catenin modulation by Dkk1 and SB216763 on the squamous metaplasia marker involucrin and the epithelial marker E‐cadherin. Immunofluorescent staining after 24 days showed increased involucrin expression when stimulating the β‐catenin pathway with SB216763. On the other hand, Dkk1 decreased involucrin expression (Fig. 6A). Involucrin mRNA expression increased in SB216763‐treated cells compared to Dkk1‐treated cells (1.52 ± 0.19 vs 0.61 ± 0.04 fold) and control condition at 1 ± 0.17 (*n* = 6 from three different lungs, Fig. 6B). Similarly, SB216763 increased significantly involucrin protein levels to 1.45 ± 0.12 fold compared to control 1 ± 0.09 fold and Dkk1 0.8 ± 0.082 fold (day 24 of culture; Fig. 6C, *n* = 4 from two different lungs).

Opposite effects were observed with the expression of the epithelial marker E‐cadherin (Fig. 6). Quantitative RT‐PCR did not show changes in the E‐cadherin mRNA expression between control and cells treated with SB216763 or Dkk1 (1 ± 0.09 vs 1.1 ± 0.2 vs 1.5 ± 0.4; *n* = 4 from two different lungs). On the other hand, SB216763 decreased the protein expression of E‐cadherin on day 24 (0.6 ± 0.82 fold) compared to control conditions (1 ± 0.09 fold) with Dkk1 of (0.87 ± 0.72 fold; *n* = 6 from three different lungs). Effects of SB216763 and Dkk1 on the structure of the airway epithelium are demonstrated in Figure S1.

In conclusion, these experiments suggest that the fate of airway epithelial cell differentiation depends on appropriate Wnt signaling. Stimulation of the canonical Wnt pathway with SB216763 drives the airway epithelium into a squamous cell phenotype as seen by increased involucrin and decreased E‐cadherin expression.

## Discussion

**Figure 7:**
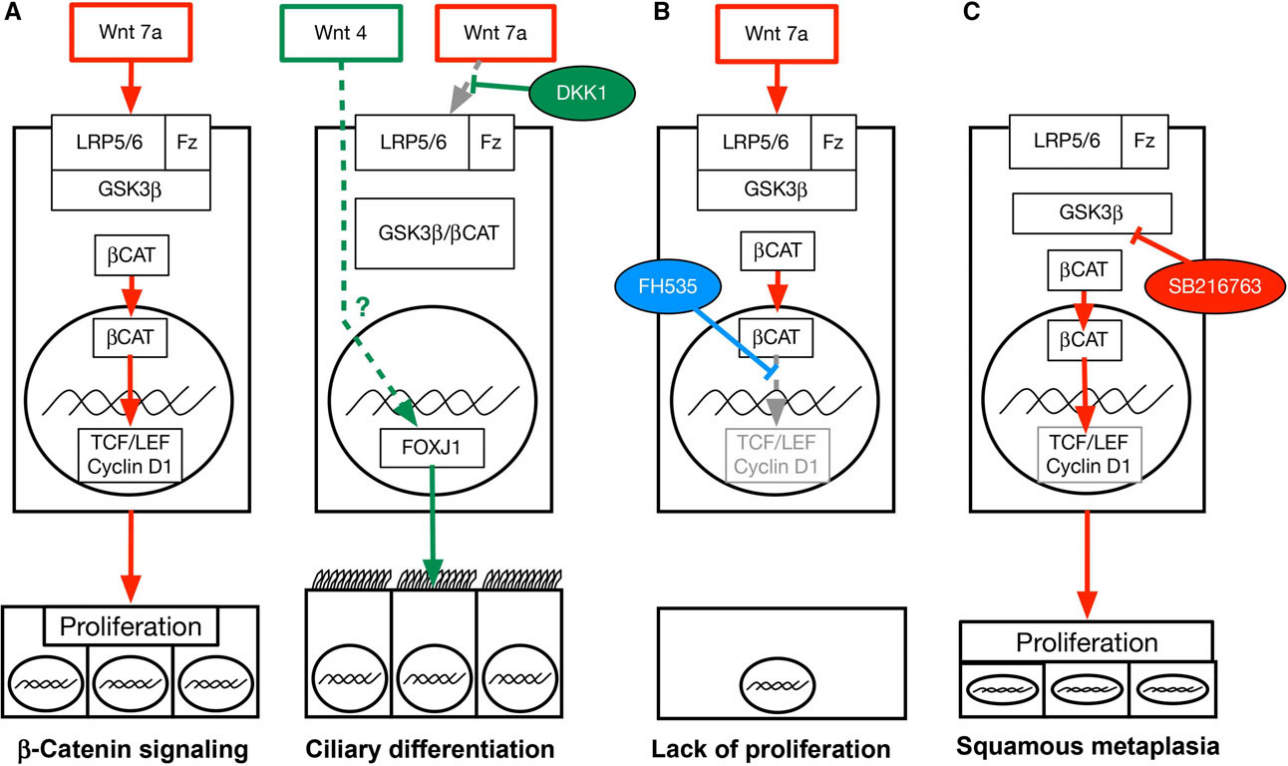
Schematic to illustrate model of Wnt pathway. Figure illustrates the suggested mechanism of Wnt signaling discussed in the text. (A) Phase of proliferation of airway epithelial cells with canonical signaling by Wnt7A binding Fz receptors, leading to inactivation of GSK3β and increase of β‐catenin content. Increased Dkk1 inhibits Wnt7a signaling, freeing GSK3β that binds β‐catenin. Wnt4 instead enhances FOXJ1 expression and ciliary differentiation. The mechanism of this effect is unclear as indicated with a dotted line and question mark in the figure. (B) FH535 inhibits β‐catenin interaction with TCF/LEF, blocking proliferation. (C). SB216367 blocks GSK3β, leading to unopposed β‐catenin signaling that causes increased proliferation and squamous metaplasia.

Our data suggest an important role for Wnt signaling during differentiation of the airway epithelium to a ciliated cell phenotype. We found that continuous enhancement of β‐catenin signaling by inhibition of GSK3β inhibits ciliogenesis and promotes squamous metaplasia, whereas decreasing β‐catenin pathway signaling with Dkk1 enhances differentiation of the ciliated cell population. Blocking signaling with FH535 showed that β‐catenin is essential during the proliferation but not during the differentiation phase. These data suggest the need for a switch from β‐catenin‐dependent to β‐catenin‐independent Wnt signaling during airway epithelial cell differentiation (Fig. 7).

Wnt signaling is an important regulator of proliferation and differentiation processes 18. Overwhelming canonical Wnt signaling is associated with uncontrolled proliferation, leading to neoplastic behavior. This is classically shown by the APC gene defect that leads to adenomatous polyposis of the colon based on increased cellular β‐catenin content 48. However, the role of β‐catenin in the differentiation of ciliated cells is currently unclear. Overexpression of β‐catenin in mouse airway epithelial cells has been shown to increase the number of ciliated cells 49, to decrease them 50, or not to change them 51. Another report describes the need of β‐catenin for early fate determination of progenitor cells, but not at a later time when ciliogenesis occurs 52. In our hands, using primary human airway epithelial cells at the ALI, continuous stimulation of β‐catenin signaling during differentiation leads to a significant decrease of FOXJ1 expression, associated with a decreased ciliated cell population (Figs 5 and 6).

Exploring the influence of Wnt signaling during differentiation, we found an early phase with increased expression of canonical Wnt signaling element expressions, including Cyclin D1 followed by a second phase characterized by decreased expression of canonical Wnt signaling elements but a dramatic increase of the ciliated cell fate marker FOXJ1 (Fig. 2A). These findings show that an early phase of proliferation is followed by a phase of differentiation, with an associated switch in Wnt signaling. Thus, our data support the necessity for decreased β‐catenin signaling to achieve ciliated cell differentiation 53.

To further evaluate the importance of β‐catenin for early fate determination as previously described 52, we blocked β‐catenin signaling with FH535 and showed that initial β‐catenin signaling is essential for the propagation of airway epithelial cells (Fig. 3). However, it remains unclear whether β‐catenin is needed only for proliferation or also for fate determination. If the role of β‐catenin was mainly related to fate determination, inhibition of β‐catenin during the proliferation phase would be expected to keep the cells in an undifferentiated state. This does not happen. In fact, some cells die within a few days (Fig. 3), pointing to an important proliferative role of β‐catenin signaling.

One could argue that the FH535 effect is based on cell toxicity. This is unlikely, as the addition of the same concentration of FH535 restricted to the differentiation phase (Fig. 3D) did not affect cell viability. Additionally, staining cilia to determine ciliation (Fig. 3E) did not show any obvious effects on cell viability. Together, these results suggest that β‐catenin is essential for proliferation 54.

FH535 is known to affect PPARγ signaling by preventing the recruitment of β‐catenin to PPAR‐γ and thus inhibiting β‐catenin/PPAR‐γ interaction 44. Interactions between Wnt and PPARγ signaling have been described in different lung compartments. Upregulation of Wnt signaling and downregulation of PPARγ signaling affect differentiation of interstitial fibroblast in the human alveolar compartment 55 and treatment with PPARγ agonist prevent Wnt‐associated, hypoxia‐induced neonatal lung injury 56. Additionally, PPARγ signaling is known to affect differentiation of the airway epithelium 57. Furthermore, the importance of Wnt signaling in neonatal lung maturation is well known 18, 24, 27, 58. Based on this, we cannot exclude that part of the observed effect of FH535 may be related to PPARγ signaling, but the interaction of the two pathways is complex and needs further investigation.

The expression pattern of Wnt7a and Wnt4 (Fig. 2A) showed clear changes of expression at the interface of proliferation and differentiation, indicating their possible role in this process. Wnt7a was highly expressed during proliferation and decreased during differentiation, whereas Wnt4 expression showed an inverse pattern. Wnt7a is known to signal canonically 59 and has been described to stimulate proliferation of skeletal muscle 60 and human hair follicular stem cells 61. It has also recently been shown that human airway basal cells express only Wnt7a whereas mouse basal cells also express Wnt3a, Wnt5b, and Wnt9a 62. These findings let us speculate that Wnt7a plays a major proliferative role in human basal cells.

Wnt4 is classically described as a noncanonical signaling molecule via activation of mitogen‐activated protein kinase 8 63 or via its interaction with the Fz‐6 receptor 5, 63, 64, 65. On the other hand, Wnt4 has also been described to play a role in canonical signaling 5, 66, 67, 68. The significant increase of Wnt4 in our cell system occurs simultaneously with increased expression of Dkk1. This coexpression indicates that Dkk1 can inhibit β‐catenin‐dependent signaling through Wnt7a and Wnt4 and stimulate differentiation through a β‐catenin‐independent pathway.

Inhibition of GSK3β with SB216763 blocks intracellular β‐catenin degradation and stimulates canonical signaling, independent of Wnt signals. Therefore, Dkk1 cannot inhibit canonical signaling in the presence of SB216763. The fact that development of cilia does not occur under these circumstances underlines the importance of the inhibition of the β‐catenin signal for ciliated cell development. This also implies that activation of the noncanonical pathway by Dkk1, independent of its blocking effect on the canonical Wnt pathway, cannot rescue the ciliated cell population.

Wnt signaling has been shown to be important for the regulation of stem cell proliferation 60. Coculture experiments of rat mesenchymal stem cells at the bottom of wells with rat tracheal epithelial cells on Transwell filters sharing the same medium showed a differentiation of the stem cells into epithelial cells 69 with expression of occludin, cytokeratin 18 70, and cystic fibrosis transmembrane conductance regulator or CFTR 71. These are markers of ciliated cells. Inhibiting GSK3β with lithium chloride (LiCl) led to a decreased expression of cytokeratin and CFTR in this system, whereas the exposure to Dkk1 increased both of these airway epithelial markers.

Stimulation of canonical Wnt signaling by overexpressing β‐catenin leads to squamous metaplasia in a mouse model 72. Similar results have been described in chronic rhinosinusitis with nasal polyps. The canonical Wnt pathway is activated in nasal polyps and leads to the formation of an abnormal epithelium with compromised adherents junctions, absent ciliogenesis, and impaired PCP signaling 73. We show here that stimulation of the canonical Wnt pathway by inhibition of GSK3β leads to similar features in human airway epithelial cells with increased expression of involucrin, a marker of squamous metaplasia 74, a decrease in the expression of E‐cadherin, a maker for epithelial structure 75, and significant reduction of the ciliated cell population.

In conclusion, our data suggest a biphasic Wnt signaling pattern during airway epithelial repair with a switch of signaling from an initial β‐catenin‐dependent to a β‐catenin‐independent pattern before differentiation of ciliated cells occurs. Permanent stimulation of the β‐catenin signaling via GSK3β inhibition leads to squamous metaplasia without differentiation of ciliated cells whereas suppression of canonical signals with Dkk1 enhances the ciliated cell population, supporting the importance of a switch in Wnt signaling.

## Author contributions

AS: Wrote the grant and initiated the project. Planning, executing and analysis of experiments. Making figures and writing drafts of manuscript. JS: Planning, executing and analysis of experiments. Writing and review manuscript. LN: Executing and analysis of experiments. NB: Executing experiments. Making figure and review manuscript. NF: Mentoring project and review manuscript. MS: Mentoring project and review manuscript.

## Supporting information


**Fig. S1.** Confocal imaging of the squamous pattern of cultures treated with SB216763.
