# Supplementary figures and images for: Modulation of Wnt signaling is essential for the differentiation of ciliated epithelial cells in human airways
Source: FEBS Lett. 2017 Oct 10;591(21):3493–506. doi: 10.1002/1873-3468.12851 (PMC5683904; doi:10.1002/1873-3468.12851)

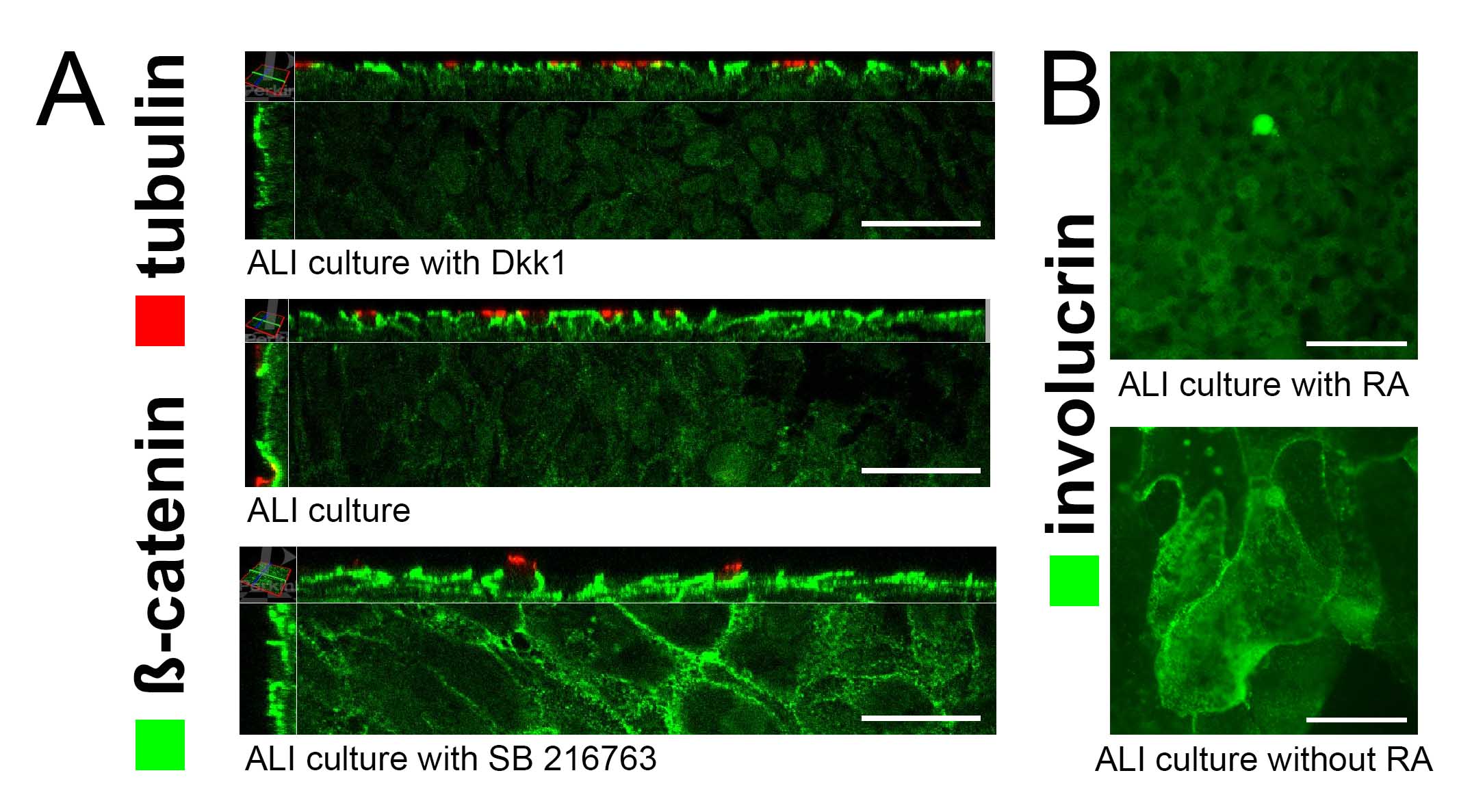

Supplement: Supplementary file 1 — Fig. S1. Confocal imaging of the squamous pattern of cultures treated with SB216763. [file FEB2-591-3493-s001.jpg]
